# Development of Chitosan Microspheres through a Green Dual Crosslinking Strategy Based on Tripolyphosphate and Vanillin

**DOI:** 10.3390/molecules26082325

**Published:** 2021-04-16

**Authors:** Rodolpho Fagundes Correa, Giovana Colucci, Noureddine Halla, João Alves Pinto, Arantzazu Santamaria-Echart, Silvia Priscila Blanco, Isabel Patrícia Fernandes, Maria Filomena Barreiro

**Affiliations:** 1 Centro de Investigação de Montanha (CIMO), Campus de Santa Apolónia, Instituto Politécnico de Bragança, 5300-253 Bragança, Portugal; rodolphocorrea@alunos.utfpr.edu.br (R.F.C.); giovana.colucci@ipb.pt (G.C.); jpinto@ipb.pt (J.A.P.); ipmf@ipb.pt (I.P.F.); 2 Campus Londrina, Universidade Tecnológica Federal do Paraná, Av. dos Pioneiros, 3131-Jardim Morumbi, 86036-370 Londrina, Brazil; silviablanco@utfpr.edu.br; 3 Laboratory of Biotoxicology, Pharmacognosy and Biological Recovery of Plants, Department of Biology, Faculty of Sciences, University of Saida, Saïda 20000, Algeria; noureddine.halla@univ-saida.dz

**Keywords:** chitosan microspheres, dual crosslinking strategies, green crosslinker, tripolyphosphate, vanillin

## Abstract

Microencapsulation procedures have recently focused attention on designing novel microspheres via green synthesis strategies. The use of chitosan (CS) as an encapsulating material has increased interest due to its unique bioactive properties and the various crosslinking possibilities offered by their functional groups. The consolidation of the microspheres by physical crosslinking using sodium tripolyphosphate (TPP) combined with chemical crosslinking using vanillin (VA) open new opportunities in the framework of green dual crosslinking strategies. The developed strategy, a straightforward technique based on an aqueous medium avoiding complex separation/washing steps, offers advantages over the processes based on VA, mostly using water-in-oil emulsion approaches. Thus, in this work, the combination of TPP crosslinking (3, 5, and 10 wt.%) via spray-coagulation technique with two VA crosslinking methods (in situ and post-treatment using 1 wt.% VA) were employed in the preparation of microspheres. The microspheres were characterized concerning morphology, particle size, physicochemical properties, thermal stability, and swelling behavior. Results revealed that the combination of 5 wt.% TPP with in situ VA crosslinking led to microspheres with promising properties, being an attractive alternative for natural bioactives encapsulation due to the green connotations associated with the process.

## 1. Introduction

Microencapsulation consists of the isolation and protection of active principles (e.g., solids, liquids, or gaseous materials) from the surrounding medium within microscopic-sized shells or particles (1 to 1000 μm), usually made by polymeric materials [1,2]. This technology presents several advantages to enable active principles’ applicability. Microencapsulation protects unstable and sensitive compounds, masks undesired tastes and odors, improves solubility or dispersibility facilitating the processability, and the design of controlled and target release delivery systems [1,2,3]. In this way, microencapsulation is employed in several application fields, including food, agriculture, chemicals, pharmaceuticals, and cosmetics [2,4]. The broad range of possibilities to design microspheres, together with the recent focus on green strategies, has evolved to solutions involving biopolymers [4,5,6] and avoiding toxic chemicals [7,8].

Chitosan (CS) is presented as an attractive material for microsphere’s preparation [9]. Chitosan results from chitin’s deacetylation, a renewable biopolymer extracted from crustaceans. Chitosan is composed by d-glucosamine (2-amino-2-deoxy-d-glucose) and *N*-acetyl-d-glucosamine (2-acetamido-2-deoxy-d-glucose) units linked by β (1→4) glycosidic bonds. The polymer’s deacetylation degree (DD) needs to be higher than 60% for being considered chitosan, of which the molecular weight typically ranges between 50 and 2000 kDa [9]. Chitosan presents bioactive features, namely, antibacterial, antifungal, antioxidant, and anticancer properties, biodegradability, and non-toxicity. It can be used in various forms including as powder, hydrogels, films, membranes, and fibers [10], finding uses in diverse fields such as biomedicine, cosmetology, agriculture, food, and pharmaceutics. Nevertheless, chitosan’s use can be restricted due to inherent limitations, namely the solubility in acidic medium (pH < 6.3) that is dependent on amine group’s protonation [11,12], the brittleness, and the instability in aqueous and physiological environments required for food, pharmaceutical, and medical applications.

Crosslinking trough physical and chemical strategies [11,13] has gained attention to develop useful chitosan-based materials. In this context, new strategies using natural, non-toxic, and sustainable crosslinking agents (both physical and chemical) as promising substitutes to the hazardous chemical counterparts is a rising scientific research thematic [14,15,16,17,18]. Ionic or physical crosslinking results from electrostatic interactions between appositively charged sites of the crosslinking agent (polyanion) and the chitosan (positively amino groups in acidic media). Among others, sodium tripolyphosphate (TPP) is recognized as a non-toxic polyanion, classified by the Food and Drug Administration (FDA) as a Generally Recognized as a Safe Substance (GRAS), supporting its use in a vast domain of applications [19]. TPP is extensively applied to biopolymer’s crosslinking [18], including to produce nano- and microparticles for controlled delivery of active cores through different encapsulation methods [16,17,20,21,22].

Covalent or chemical crosslinking is based on chitosan’s primary and secondary hydroxyls (-OH), and non-protonated and protonated amine functional groups (-NH_2_ and -NH_3_^+^), which constitute reactive sites to link with crosslinking agents with at least two reactive sites [18]. The most commonly used agents include glutaraldehyde (and derivatives) [23,24] and genipin [25]. However, the reported cytotoxicity of their outcome structures led to the progressive discharge of their use [26], motivating studies to find greener alternatives.

Vanillin (VA) is a naturally occurring compound obtained from the beans or pods of the tropical vanilla orchid, being also obtained by synthetic routes, e.g., from lignin [27]. VA exhibits bioactive properties justifying its application as one of the most popular substances in the field of flavoring additives, food preservatives, perfumery, cosmetics, and drugs [28]. VA, 4-hydroxy-3- methoxybenzaldehyde, is classified as a bio-based crosslinking agent due to the presence of an aldehyde functional group (-CHO), together with a hydroxyl group (-OH). Typically, it is reported that the aldehyde group reacts with chitosan (-NH_2_ groups) leading to a Schiff base (N=CH) formation [14]. Nevertheless, other works also reported the acetalization reaction induced by the conjugative effect of VA’s benzene ring, which can passivate the nucleophilic addition reaction of the aldehyde group, promoting the hemiacetal intermediate to favor the acetalization reaction [29].

In this work, chitosan-based microspheres were prepared using a dual crosslinking strategy, namely by combining two sequential steps of physical and chemical crosslinking. This approach has advantages over the ones using only VA, which are mostly based on water-in-oil emulsion approaches. Namely, it is a straightforward technique based on aqueous medium avoiding complex separation/washing steps, which is an advantage for an industrial implementation of the process. Firstly, physical crosslinking with TPP was applied using the spray-coagulation technique. Thereafter, the prepared microspheres were chemically crosslinked with VA using two alternative procedures. By the in situ method, microspheres were crosslinked directly in the TPP coagulation bath by VA addition to the solution bath. By the post-treatment method, the consolidated microspheres were recovered from the coagulation bath by vacuum filtration, then chemically crosslinked with VA using a freshly prepared solution. Microspheres were prepared using different TPP contents (3, 5, and 10 wt.%) combined with 1 wt.% VA, according to the two described methodologies. The systems were characterized in terms of morphology, particle size, physicochemical properties, thermal stability, and swelling behavior aiming at establishing the best solutions for future microencapsulation of natural bioactives, thus achieving sustainable and green products. Considering the novelty of the present work, although each crosslinking method (i.e., using tripolyphosphate or vanillin) has already been reported, to the best of our knowledge, in situ and sequential dual crosslinking methods of chitosan microspheres using the combined processes have not been investigated before.

## 2. Results and Discussion

### 2.1. Microspheres’ Morphology during Formation

**Figure 1:**
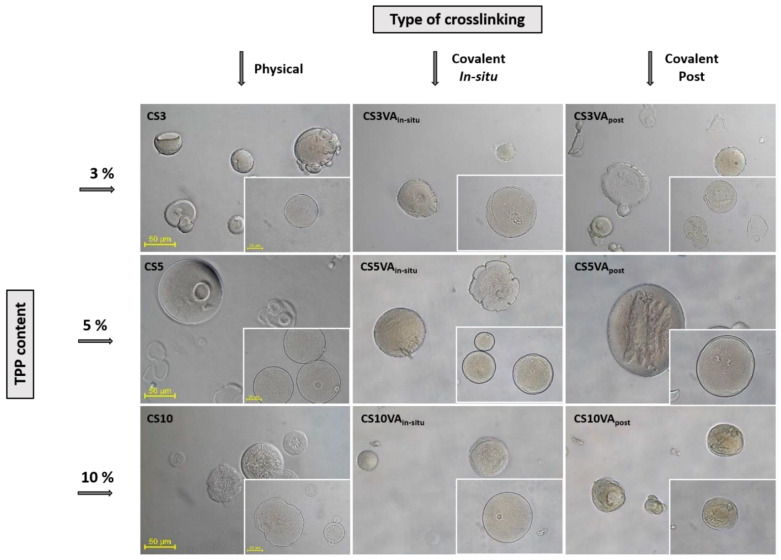
OM images of the microspheres obtained after the applied crosslinking steps at 200× magnifications (scale bar 50 µm). In the inset, the corresponding magnification is 400× (scale bar 25 µm).

Figure 1 shows the optical microscopy (OM) images of the microspheres during the preparation process. Table 1 summarizes the results obtained for the particle size. The particle size was determined after the two defined steps, namely the ionic and chemical crosslinking steps. Based on the obtained results, a statistical analysis was conducted considering the 3 TPP contents’ groups to determine the *p*-value through ANOVA analysis. The significance for both ionic and covalent crosslinking strategies was analyzed with a confidence interval of 95% (α of 0.05), obtaining *p*-values of 1.99·10^−5^ and 1.73·10^−^4, respectively. The determined *p*-values, which the defined α values are below, evidenced that the variations in TPP content were significant.

The morphology of the base microspheres, i.e., the microspheres after the ionic crosslinking step, was influenced by the TPP concentration, where a better consolidation of the particles can be observed as the TPP concentration increased, which was associated to a more extensive level of ionic crosslinking. When 3% of TPP was employed (CS3), the microspheres exhibited a more fragile and unregular shape, showing the lowest values of particle size, evidencing that this TPP concentration was not enough to provide an effective crosslinking [19]. As the TPP concentration increased, microspheres become more spherical and better consolidated, which was accompanied by an increase in the particle size for the particles produced with 5% TPP. Nevertheless, at high TPP concentration (10%), the effect on particle size was inverted, i.e., the higher crosslinking level induced the formation of denser microspheres whose hydrodynamic volume was reduced [30].

Concerning the chemical crosslinking effect, both strategies provided a reinforcement effect on the microspheres, perceptible by the appearance of more defined particles and, in general, of smaller sizes, in comparison with their precursors after the ionic crosslinking step. The observed size reduction can be related with an effective VA reticulation that favored particle’s consolidation. Comparing the two strategies, it was observed that the post-treatment method led to a loss of the particle’s sphericity, and to the development of rough surfaces. This observation could be associated with the dehydration and mechanical stress imparted to the microspheres during the applied filtration stage preceding the chemical crosslinking step.

### 2.2. Microspheres’ FTIR Structural Analysis

**Figure 2:**
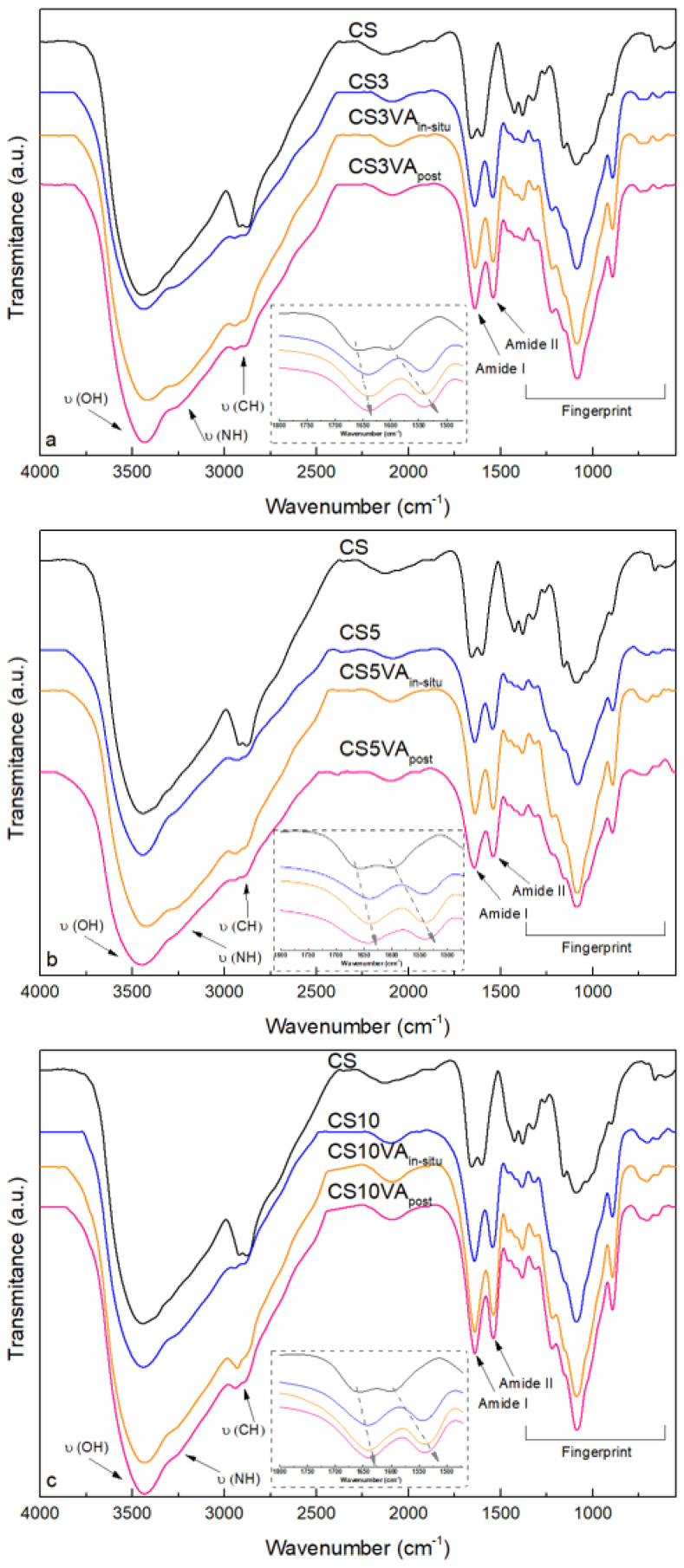
FTIR spectra of CS (black) and microspheres prepared by TPP physical crosslinking (blue), in situ (orange), and post-treatment (pink) methods. Series of (**a**) 3%, (**b**) 5%, and (**c**) 10% TPP. In the insets, the amide I and amide II regions are shown.

The spectra of the original base chitosan (CS), the microspheres after the ionic crosslinking step (CS3, CS5 and CS10), the microspheres after in situ VA crosslinking (CS3VA_in situ_, CS5_in situ_, and CS10_in situ_), and the microspheres after the post-treatment VA crosslinking (CS3VA_post_, CS5_post_, and CS10_post_) are shown in Figure 2. The relevance of the dual crosslinking over single step strategies should be highlighted, particularly to improve the resistance of chitosan dissolution in acidic pH media. The TPP treatment implies a physical action, strong enough to confer the particle conformation, but can result in structures sensitive to acidic pH media. The combination of TPP with VA crosslinking reinforces this resistance through the formation of covalent crosslinking points.

Base chitosan presented a broad region from 3865 to 2376 cm^−1^ with several vibrations, which include the stretching of hydroxyl (O-H) and amine (N-H) groups centered at 3440 and 3294 cm^−1^, respectively, and the bands at 2916 and 2877 cm^−1^, associated to C-H stretching vibrations [31]. In the amide I and II region (1774–1234 cm^−1^), the stretching vibration at 1658 cm^−1^ is attributed to the carbonyl (C=O) of the acetamido groups, the band at 1604 cm^−1^ to the angular deformation of N-H groups of the acetamido and amino groups, and the band at 1326 cm^−1^ to the C-N groups [32]. The peaks at 1380 and 1427 cm^−1^ are attributed to the C-H symmetrical deformation vibrations. The fingerprint in the glycosidic region (1234–740 cm^−1^) included the glycosidic C-O-C and C-O stretching vibrations at 1157 and 1087 cm^−1^, respectively, and the band at 894 cm^−1^ is associated to the pyranose ring [33].

Analyzing microsphere’s spectra, band displacements were observed when compared with the original CS spectra, an effect influenced by the applied chemical crosslinking method (in situ or post-treatment). It was observed that the band associated with the NH groups of chitosan was displaced from 3294 to 3271 cm^−1^ in the produced microspheres. Similarly, the band attributed to the O-H stretching vibration of CS, assigned at 3440 cm^−1^, barely shifted to lower wavenumbers after the TPP crosslinking, while its broadening was slightly enhanced after the VA crosslinking, evidencing the higher interaction effect derived from the covalent crosslinking approach.

The amide I and II regions of the spectra are included in the insets of Figure 2. Regarding the amide I region, and in comparison with CS, the carbonyl groups vibration assigned at 1658 cm^−1^, was dislocated to lower wavenumbers (1643 cm^−1^) in the microspheres, influenced by both crosslinking steps. In particular, derived from the chemical crosslinking a new imine group (C=N, Schiff base) was formed upon the condensation reaction between the free CS amine groups (-NH_2_) and the VA aldehyde groups (-CHO), reflected in a stretching vibration around 1640 cm^−1^ [15,28,34]. The electrostatic forces generated between chitosan (-NH_3_^+^) and TPP (-PO^−^) by the physical crosslinking, caused a steric effect intensifying the intra- over the inter-hydrogen bonding of the amide C=O and N-H groups, lengthening the bonding and resulting also in the lowering of these absorption frequencies [17,35]. The CS-TPP interactions also contributed to displace the N-H (amide II) vibration from 1596 to 1542 cm^−1^ in the microspheres’ spectra. Noteworthily, a band’s intensification was observed when using the in situ method (in comparison with the post method), and when decreasing TPP content (from 10 to 3%). This last point indicated a lower TPP crosslinking, increasing the free NH_3_^+^ groups, which remained available for the next covalent step. Particularly, in the case of 5% TPP, the balance between the achieved physically crosslinked and the remaining free amino groups provided a suitable platform to promote the later VA chemical crosslinking, which is reflected in the band’s intensification, mainly when using the in situ method (in comparison with the post method).

In the polysaccharide fingerprint region, the appearance of a new strong band at 1218 cm^−1^ in the microsphere spectra was attributed to the P=O groups of the TPP, while the bands at 1087 cm^−1^ and 894 cm^−1^ were sharpened due to the presence of the PO_3_ and P-O-P groups of the TPP crosslinking agent.

### 2.3. Microspheres’ Thermal Analysis

**Figure 3:**
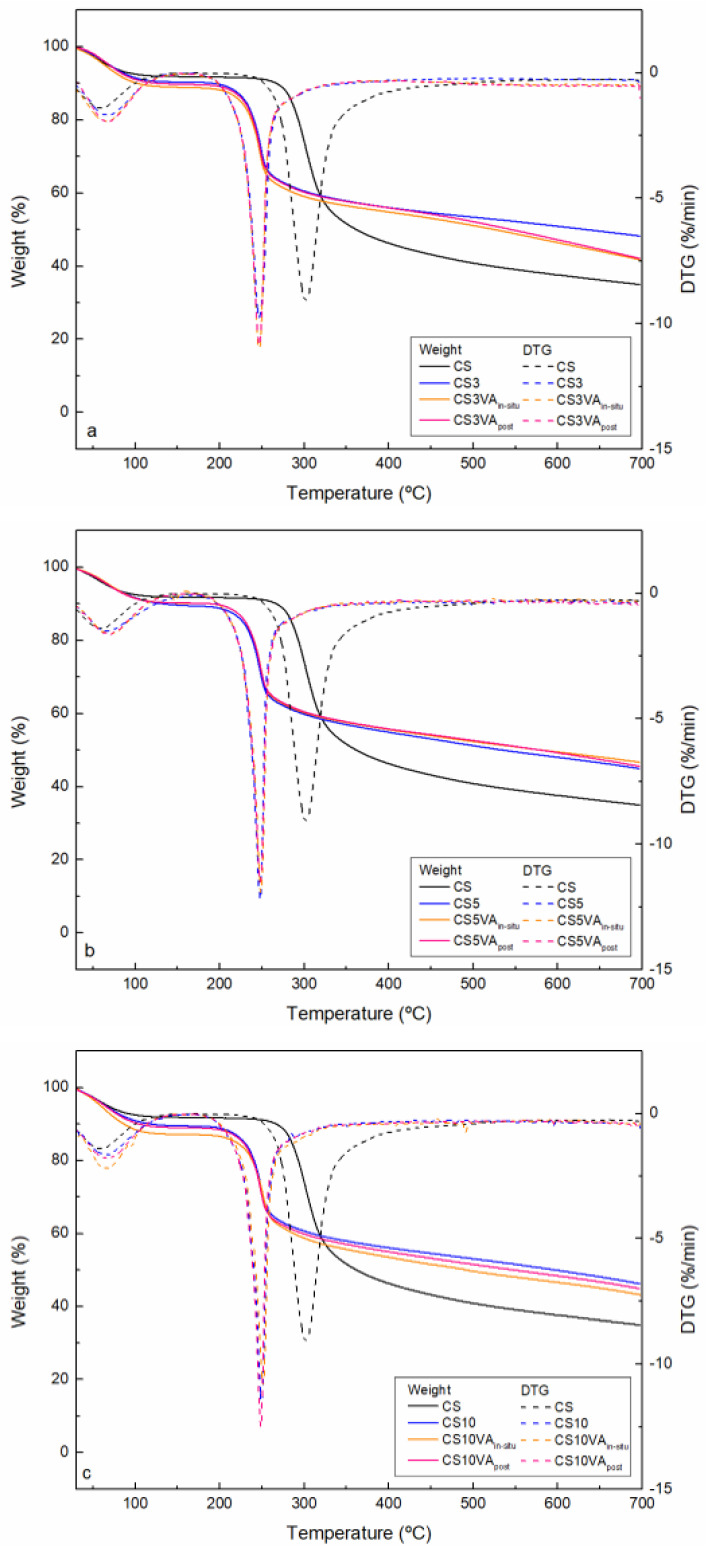
TG and DTG curves of chitosan (black) and microspheres prepared by TPP physical crosslinking (blue), in situ (orange), and post-treatment (pink) methods. Series of (**a**) 3%, (**b**) 5%, and (**c**) 10% TPP.

The thermal stability of CS and of the obtained microspheres (in dry state) was analyzed by a thermogravimetric analysis (TG). The TG and the samples’ derivative DTG curves are shown in Figure 3. The obtained results are summarized in Table 2. Analyzing the CS thermogram, two main degradations steps were observed, namely in the ranges 30–150 and 230–408 °C. The obtained final residue was 32 wt.%. The first weight loss step (8.2 wt.%) is associated to the evaporation of the water physically absorbed to chitosan [32]. The second transition (46.2 wt.%), with a maximum degradation temperature of 302, corresponds to the chitosan’s depolymerization through the cleavage of the glycosidic linkages and degradation of monomeric units [36].

For the microspheres, two degradation steps were also observed. In general, final microspheres. i.e., microspheres subjected to TPP and VA crosslinking, evidenced an increase in the maximum water evaporation temperature, suggesting that the crosslinking strategies favored a stronger water entrapment into the microspheres’ structure, thus requiring higher temperatures to be released. Moreover, and especially in the case of the 5%TPP samples subjected to chemical crosslinking (CS5VA_in situ_ and CS5VA_post_), a higher maximum water evaporation temperature was observed (71 °C). This fact was accompanied by the lower adsorbed water (9.7 and 9.8%, respectively), comparatively with the base microspheres (CS5). As reported by Tomadoni and co-workers [34], the effective CS-VA crosslinking can be evidenced by an increase in the initial water evaporation temperature, and in the decrease of the water absorption capacity of the material (a more hydrophobic character results due to the crosslinking). In this case, a higher CS-VA Schiff base (C=N) and hydrogen bonding formation occurred between CS and VA, as previously corroborated by the FTIR results.

Concerning the second degradation step, a decrease in the weight loss (W_loss2_) values was observed for all the series, as well as a displacement to lower temperatures. As a consequence, the final residue was higher, a fact that can be associated to a fraction of the material being more resistant to thermal degradation, and thus, to an improved crosslinking effect. In the case of using 3 and 10% of TPP, the residue of the ionically crosslinked microspheres was higher compared to the respective covalently crosslinked microspheres, which pointed out a significant contribution of the physical crosslinking with TPP over the chemical crosslinking. When using 5% TPP, a better balance between ionic and covalent crosslinking seems to happened, which is evidenced by the similar residue obtained for the three samples of the series (CS5, CS5VA_in situ_, and CS5VA_post_).

### 2.4. Microspheres’ Swelling Tests

**Figure 4:**
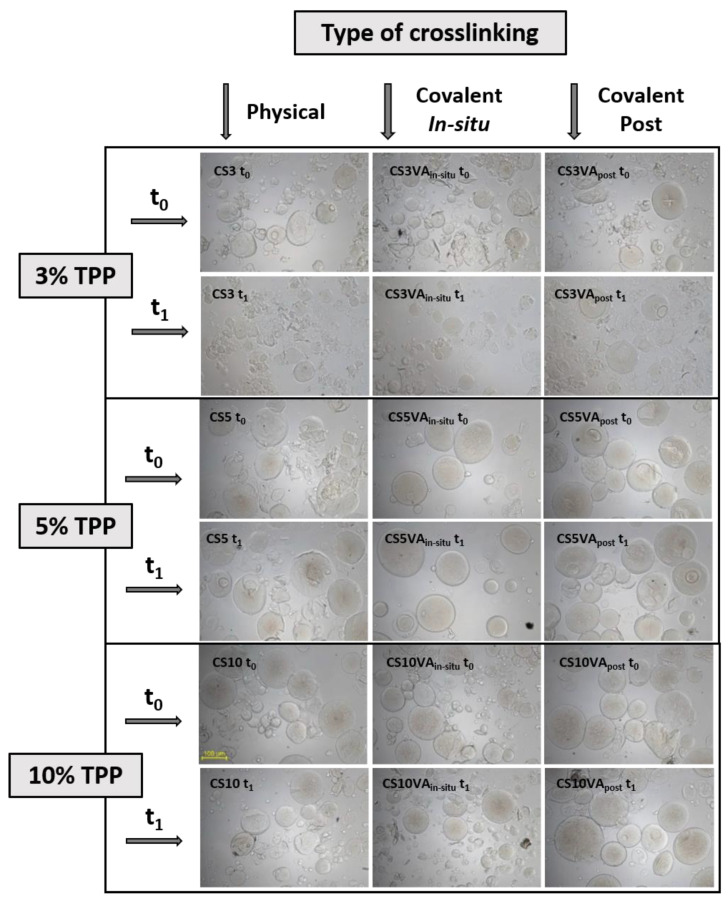
OM images of microspheres in swelling tests after 1 h (t_0_) and 1 day (t_1_) at 100× magnification.

The effectiveness of the crosslinking steps on the prepared microspheres was analyzed by studying the swelling behavior in acidic medium (pH 3.2). If crosslinking was not effective, microspheres will tend to disrupt due to the good solubility of chitosan in acidic medium. Figure 4 shows the images obtained by OM for the analyzed times: 1 h (t_0_) and 1 day (t_1_).

Analyzing the effect of ionic crosslinking with TPP, it was generally observed that microspheres were not stable in acidic medium, resulting in a pronounced disruption. Nevertheless, the progressive increase of TPP content reduced this effect, leading to lower microspheres disaggregation, both for t_0_ and t_1_. Moreover, 5 and 10% TPP contents presented similar swelling behavior, while for 3% TPP, the disruption of the microspheres was highly evident.

When microspheres were subjected to the chemical crosslinking step with VA, the stability in acid medium increased, although for 3% TPP, the improvement was very modest, with no significative difference between both methods (in situ or post treatment). For the 5 and 10% series, the chemical crosslinking effect imparted a positive effect, decreasing the disaggregation of the microspheres. This effect was particularly evident for the 5% series, where the dual crosslinked microspheres, namely CS5VA_in situ_ and CS5VA_post_, maintained their shape and appearance without evident disruption phenomena, in comparison to the physically crosslinked counterparts (CS5), which resulted in partial disruption. Specifically, CS5VA_in situ_ sample should be highlighted since the shape of the microspheres was preserved even after 1 day in contact with the acidic medium. Moreover, no extra swelling variations from t_0_ to t_1_ were observed, resulting in structures stronger than CS5_post_, and particularly than CS5. This could only be justified by the occurrence of an effective chemical crosslinking with VA. Thus, in alignment with previous results, the more effective crosslinking strategy was the in situ strategy, and the composition leading to the most promising balance between ionic and chemical crosslinking corresponds to the series using 5% TTP.

In summary, the use of 3% TPP was not enough to provide sufficient ionic crosslinking to proceed with the subsequent chemical crosslinking stage, as can be seen by the results obtained with the CS3 microspheres. In the case of using 10% TPP, a high extent of the ionic crosslink was achieved hindering the later VA chemical crosslinking stage. This can be perceived by the results obtained with the CS10VA_in situ_ and CS10VA_post_ samples. The predominance of the ionic crosslinking justifies the higher disruption of the microspheres, especially after 1 day (t_1_), a behavior similar to the one observed for the base microspheres (CS10). For the case of 5% TPP, the formation of consolidated microspheres due to ionic crosslinking with TPP, but holding enough free amine groups for the later VA crosslinking, favored when VA was directly added to the TPP coagulation bath (in situ method), resulting in a synergistic crosslinking approach.

### 2.5. Microspheres’ SEM Analysis

**Figure 5:**
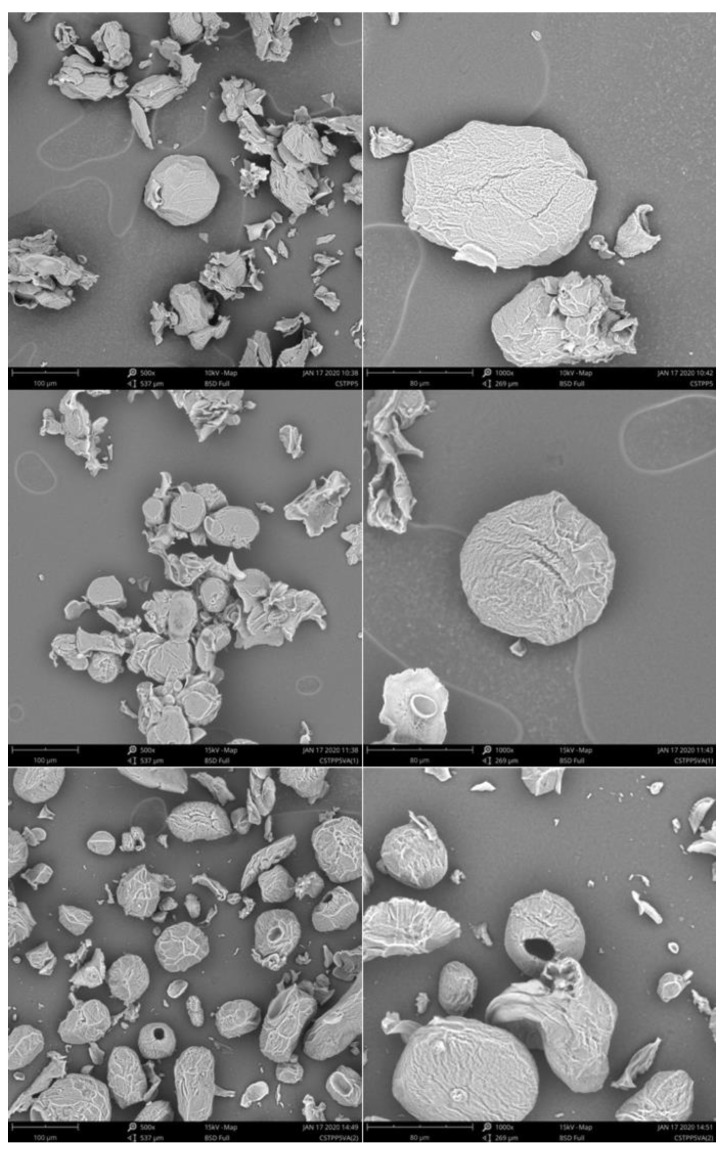
SEM images of 5% TPP microspheres’ series at 320×, 500×, and 1000× magnifications. CS5, CS5VA_in situ_, and CS5VA_post_ in the top, middle, and bottom rows, respectively.

The microspheres corresponding to the 5% TPP series (CS5, CS5VA_in situ_, and CS5VA_post_), i.e., the samples holding the most effective crosslinking due to a more equilibrated balance between ionic and chemical crosslinking, were examined in the dry state and the obtained micrographs are shown in Figure 5. In a general way, and for all samples, consolidated structures, presenting a rough surface and a non-regular shape, were detected. The observed shrunken conformations can be associated to the mechanical stress suffered by the microspheres during the drying process (lyophilization) [37].

When comparing both VA crosslinking strategies, CS5VA_post_ microspheres exhibited a more spherical shape. Nevertheless, a frequent formation of hollow structures was observed. This fact could be associated with the post preparation strategy, where the dehydration of the microspheres during the filtration step after the ionic crosslinking may have led to the development of a superficial barrier that hinders VA penetration in the inner structure. This effect can also have been reinforced due to the used neutral pH of VA crosslinking solution. By contrast, through the in situ strategy (CS5VA_in situ_), where no intermediate drying step was used and the VA crosslinking occurred directly on the coagulation TPP bath (acidic pH), favored the microspheres swelling. In consequence, the VA penetration was facilitated, helping to promote internal crosslinking.

## 3. Materials and Methods

### 3.1. Materials

Microspheres were prepared using chitosan (CS) brand 90/200/A1 (deacetylation degree and viscosity of 91.9% and 128 mPa·s (1% at 20 °C in 1% acetic acid solution)), provided by Biolog Heppe (Landsberg, Germany). The ionic crosslinking bath was prepared using sodium tripolyphosphate purchased from Alfa Aesar (Kandel, Germany) and hydrochloric acid (HCl) supplied from Panreac (Barcelona, Spain) (as coagulation bath pH adjuster). For the covalent crosslinking, vanillin (99% of purity), acquired from Sigma-Aldrich (St. Louis MO, United States), was employed. For chitosan dissolution and swelling tests, glacial acetic acid (AcAc) purchased from JT Baker (Matsonford, PA, USA) was used. Distilled water was employed for the preparation of the dissolutions. Potassium bromide (KBr) (≥99% of purity) was used to prepare the pellets used for Fourier-transform infrared spectroscopy analysis.

### 3.2. Preparation of Microspheres

The preparation of the microspheres comprised two steps, namely ionic crosslinking and chemical crosslinking.

In the first step, CS microspheres were prepared by spray-coagulation using a Nisco Var J30 equipment (Nisco Engineering AG, Zurich, Switzerland), comprising TPP as the ionic crosslinker. The method was carried out using a 3% (*w/v*) CS solution in acidic medium (3% *v/v* AcAc solution) and a TPP coagulation bath at pH 6 (adjusted with 1 M HCl). To conduct this first step, 20 mL of the CS solution, placed in a syringe pump, was atomized into 200 mL of the coagulation bath through a 0.25 mm nozzle under a controlled flow rate of 0.1 mL/min, maintained through a pressurized N_2_ stream at 0.4 mbar. The microspheres were consolidated during the spray-coagulation process for around 3.5 h. The ionically crosslinked base CS microspheres were coded as CS3, CS5, and CS10, corresponding to 3, 5, and 10% TPP (*w/v*), respectively.

In the second step, chemical crosslinking was conducted using a 1% (*w/v*) VA solution following two alternative strategies: in situ and post-treatment methods. In both cases, the used volume of VA solution considered a NH_2,CS_:CHO_VA_ molar ratio of 1:4. In the in situ crosslinking, the VA solution was added directly to the coagulation bath at a flow rate of 5 mL/min, using a peristaltic pump, after the TPP reaction. The coagulation bath was heated to 50 °C, and the crosslinking reaction occurred during 2 h, under continuous stirring. The produced microspheres were recovered by vacuum filtration, thoroughly washed with distilled H_2_O, lyophilized, and stored for further analysis. In the post-treatment, the ionically crosslinked microspheres were recovered from the coagulation bath by vacuum filtration and washed with distilled water. The recovered microspheres were transferred to the VA solution (neutral pH), and the crosslinking reaction occurred at 50 °C for 2 h. The resultant microspheres were recovered by vacuum filtration, thoroughly washed with distilled H_2_O, lyophilized, and stored for further analysis.

**Figure 6:**
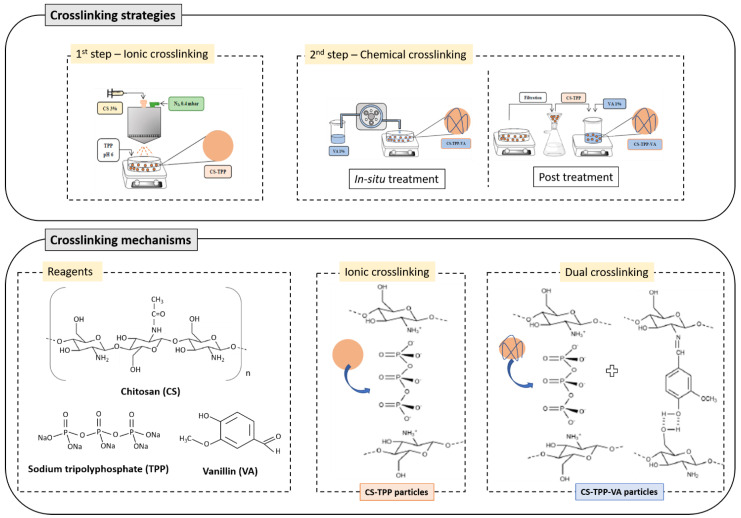
Schematic representation of the microsphere’s preparation process considering the two sequential steps, ionic crosslinking (first step) and chemical crosslinking (second step), following two strategies: in situ and post-treatment. The crosslinking mechanisms of CS with the crosslinking agents TPP and VA are also included.

A general scheme of the used procedures is presented in Figure 6. The final crosslinked CS microspheres were coded to reflect the used procedure in the second step, e.g., CS3VA_in situ_ corresponds to the CS3 microspheres submitted to in situ treatment, whereas the CS3VA_post_ corresponds to the CS3 microspheres submitted to post-treatment with VA.

### 3.3. Characterization of Microspheres

The produced microspheres were characterized by optical microscopy (OM), Fourier-transform infrared spectroscopy (FTIR), thermogravimetric analysis (TGA), and scanning electron microscopy (SEM). Moreover, swelling behavior was studied in acidic medium.

Optical microscopy was preformed using an Ni-U microscope (Nikon Eclipse Ni, Nikon Corp., Tokyo, Japan) coupled with a digital camera. The images were acquired using lens filter type A, at 100, 200, and 400× magnification with NIS-Elements Documentation software. Samples were prepared by placing a droplet of the dispersion medium containing the microspheres on a slide covered with a slip. The images were employed for determining the particle size by averaging 30 microspheres in each sample. The significance of the results variation was evaluated by ANOVA (one way) analysis for both ionic and covalent crosslinking strategies, with a confidence interval of 95% for the 3, 5, and 10% of TPP containing series.

Fourier-transformed Infrared Spectroscopy (FTIR) was performed by using an ABB Inc. FTIR, model MB3000 (Montreal QC, Canada) apparatus in transmittance mode. For that, KBr pellets with a sample concentration of 1% (*w/w*) were prepared. Spectra were recorded between 4000 and 550 cm^−1^ at a resolution of 16 cm^−1^ by co-adding 32 scans. Acquisition and data treatment was done with Horizon MB v.3.4 software.

Thermogravimetric Analysis was done using a NETZSCH TG 209F3 Tarsus (Selb, Germany) equipment. For the analysis, 6–7 mg of the sample was placed on alumina crucibles, then subjected to a dynamic run from 30 to 700 °C at a heating rate of 10 °C/min under nitrogen atmosphere (40 mL/min). TG and derivative DTG curves were obtained using Netzsch Proteus thermal analysis (v.5.2.1) software.

For the swelling tests, Microsphere’s lyophilized samples were immersed in an aqueous acidified medium (AcAc 3% (*v/v*), pH 3.2) and analyzed by OM after 1 h (t_0_) and 1 day (t_1_) period.

The morphology of the lyophilized microspheres was carried out using a Phenon Pro microscope from Phenom-World (Eindhoven, The Netherlands) scanning electron microscope (SEM). For the analysis, the lyophilized samples were placed on a carbon sheet.

## 4. Conclusions

In this work, chitosan-based microspheres were designed according to green crosslinking strategies, using a method based on two sequential steps, namely ionic crosslinking followed by chemical crosslinking. Ionic crosslinking was done with TPP and the chemical crosslinking with VA. The idea was to promote a green process based in aqueous medium to avoid the emulsion-based techniques that mostly demand laborious separation/washing steps. Thus, through the first step of ionic crosslinking, the shape was fixed to then proceed to the chemical crosslinking to reinforce the microspheres’ structure. Different TPP contents (3, 5, and 10%) were tested with a fixed concentration of VA, aiming at achieving a balanced equilibrium between ionic and chemical crosslinking. The chemical crosslinking was conducted according to two different approaches, in situ and post-treatment.

The morphology of the microspheres was determined by OM and SEM, revealing that the increase of TPP content favored the microspheres’ consolidation. Low TPP contents (3%) were not enough to consolidate the microspheres, while high contents (10%) resulted in an excess of ionic crosslinking. This excessive ionic crosslinking conditioned the later VA chemical reaction. The accessibility of the VA agent to the inner structure of the microspheres promoted a more effective crosslinking, and thus microspheres consolidation, as corroborated by the FTIR and TGA results, mainly when 5% of TPP and in situ strategy were used. In fact, in the CS5VA_in situ_ sample, the achieved balance of TPP and VA crosslinking led to microspheres that preserved the integrity better under swelling in the acidic medium. Overall, this composition led to microspheres with promising properties, which together with the proposed green dual crosslinking strategy, supports the potential of these systems to be used for natural bioactives encapsulation. Concurrently, a deeper analysis of the crosslinking degree is considered a further step to be followed in the future works aiming at understanding and designing alternative routes based on sustainable dual crosslinking strategies.

## Figures and Tables

**Table 1:** Particle size of the produced microspheres after the two applied stages (ionic and covalent crosslinking) expressed as average ± SD.

Sample	Ionic Crosslinking Particle Size (µm)	Covalent Crosslinking Particle Size (µm)
CS3	39.4 ± 13.6	-
CS3Va_in situ_	55.6 ± 19.9	54.2 ± 20.4
CS3VA_post_	45.7 ± 19.5	38.3 ± 13.8
CS5	66.9 ± 26.5	-
CS5VA_in situ_	54.9 ± 18.6	42.2 ± 14.9
CS5VA_post_	47.8 ± 14.4	55.7 ± 34.7
CS10	43.9 ± 23.4	-
CS10VA_in situ_	47.3 ± 28.2	45.2 ± 14.7
CS10VA_post_	51.0 ± 33.3	42.9 ± 15.0

**Table 2:** Maximum degradation temperature (T_p_) and weight loss (W_loss_) of the degradation steps (1 and 2) and the residue of the samples.

Sample	Degradation Step 1		Degradation Step 2		Residue (%)
	T_p1_ (°C)	W_loss1_ (%)	T_p2_ (°C)	W_loss2_ (%)	
CS	58	8.2	302	46.2	32.0
CS3	67	9.6	247	30.8	43.6
CS3VA_in situ_	64	11.1	246	30.8	37.2
CS3VA_post_	66	10.3	246	30.4	37.8
CS5	65	10.6	248	30.5	40.1
CS5VA_in situ_	71	9.7	249	31.0	42.1
CS5VA_post_	71	9.8	248	30.7	41.0
CS10	69	10.5	249	29.9	41.3
CS10VA_in situ_	65	12.8	251	29.6	37.6
CS10VA_post_	69	11.0	249	30.1	39.9

## Sample Availability

Samples are not available from the authors.
